# Novel CT-based simulation reveals anterolateral surgical approach avoids acromial collision in antegrade humeral nailing

**DOI:** 10.1007/s00068-025-03047-7

**Published:** 2026-01-13

**Authors:** Richard Arnhold, Maximilian Kern, Felix Schatzl, Christoph Epple, Franz Kralinger

**Affiliations:** 1 Department of Trauma Surgery, Klinik Ottakring, Vienna, Montleartstraße 37, 1160 Austria; 2 Doctoral Programme “Musculoskeletal and Dental Research”, Medical University of Vienna, Vienna, Spitalgasse 23, 1090 Austria; 3 Department of Radiology, Medical University of Vienna, Vienna, Spitalgasse 23, 1090 Austria; 4 Department of Orthopedics and Trauma Surgery, Klinik Floridsdorf, Vienna, Brünner Straße 68, 1210 Austria

**Keywords:** Anterolateral approach, Transdeltoid approach, Straight antegrade humeral nailing, CT-based simulation, Acromion index, Proximal humerus fracture

## Abstract

**Purpose:**

To compare implant–acromion collision risk between the transdeltoid and anterolateral surgical approaches for straight antegrade humeral nailing (SAHN) using a CT-based simulation, and to evaluate whether the Acromion Index (AI) predicts collision.

**Methods:**

Sixty-eight anonymized shoulder CTs were segmented to generate scapular and humeral surface models. A straight 8-mm nail was virtually advanced along the medullary axis under two conditions: (I) transdeltoid with 0° glenohumeral extension and (II) anterolateral with 30° extension. Two trained investigators independently repeated all simulations and recorded nail-acromion collisions. Agreement was assessed with Cohen’s κ and intraclass correlation coefficients (ICC). Collision rates were compared with paired McNemar tests. The predictive value of AI was analyzed using logistic regression and receiver-operating-characteristic (ROC) analysis.

**Results:**

Implant-acromion collision occurred in 32/68 shoulders (47.1%) with the transdeltoid approach, versus 2/68 (2.9%) with the anterolateral approach (absolute risk reduction 44%; number-needed-to-treat 2.3; *p*< 10⁻⁶). AI independently predicted transdeltoid failure (adjusted odds ratio 2.81 per +0.10; *p *= 0.011); a cut-off ≥ 0.69 yielded an AUC of 0.72 (63% sensitivity, 72% specificity). Inter-observer reliability was substantial for collision (κ 0.86) and excellent for morphometrics (ICC ≥ 0.90).

**Conclusion:**

CT-based simulation demonstrates that the anterolateral approach markedly reduces the risk of implant-acromion collision in SAHN and should therefore be considered the preferred option. However, if the transdeltoid approach is selected, preoperative AI screening with a threshold of ≥0.69 is essential, as larger acromia increase collision risk and must be factored into surgical planning.

**Level of evidence:**

Level IV – paired in-silico simulation study.

## Introduction

Proximal humerus fractures (PHF) already account for about 6% of all adult fractures in Western countries, and their incidence is climbing in step with population ageing [1, 2]. Most patients are older than 65 years and many of them show reduced bone quality due to osteoporosis, which compromises fixation strength and fracture healing [3]. As case numbers rise, it becomes increasingly important to tailor treatment to the individual patient, choosing not only between conservative care, locking-plate fixation, straight antegrade humeral nailing (SAHN) or arthroplasty, but also between the surgical approaches available for nail insertion [4].

SAHN maintains soft tissue perfusion and provides load sharing in simple two- and three-part fractures [5, 6]. The nail, however, has to reach its ideal entry point on the humeral head defined as the intersection of the intramedullary canal axis with the subchondral apex of the humeral head, situated at least 5 mm medial to the supraspinatus footprint so that the nail remains coaxial with the shaft while sparing the rotator-cuff insertion [7, 8]. Surgeons can access this entry point via two common surgical approaches. The *transdeltoid approach* splits the middle fibers of the deltoid (pars acromialis) in line with their orientation and offers a straight lateral trajectory. The *anterolateral approach* follows the raphe between the pars clavicularis and pars acromialis, which positions the working portal in most cases slightly anterolateral to the acromion [9, 10]. Only the anterolateral approach allows the humerus to be extended up to 30°, thereby exposing the ideal entry point anterior to the acromion [11, 12]. Attempting similar extension during a strict transdeltoid approach merely rotates the humeral head underneath the overlying deltoid, without increasing visual clearance. To date, no study has compared these two approaches with respect to ease of reaching the optimal entry point.

When acromial clearance is inadequate, the surgeon must choose between a lateral off-axis entry or aggressive soft tissue retraction [13]. Both choices carry penalties. A lateralized entry point, selected to bypass the acromion, drives the nail into the greater tuberosity where bone density is lower and the supraspinatus tendon inserts, increasing the risks of fixation failure and cuff injury [14–17]. Excessive retraction can damage the deltoid or rotator cuff tendons and prolong recovery [18].

The ease of reaching the entry point is dictated largely by acromial morphology [6]. The *Acromion Index (AI)*, typically measured on anteroposterior (AP) shoulder radiographs, quantifies lateral acromial overhang and has been associated with cuff tears and impingement, yet it has never been applied to guide surgical-approach selection for SAHN [19]. To address this gap, we created a computed-tomography (CT) simulation that virtually reproduces nail insertion and automatically detects implant-acromion collision.

We applied this simulation to a series of anatomically normal shoulders to (I) quantify and compare collision risk for the transdeltoid and anterolateral approaches, (II) determine whether the AI predicts collision and define a useful cut-off, and (III) explore the potential of CT-based simulation as a planning tool for patient-specific operative strategy in PHF. We hypothesized that the anterolateral approach would show a substantially lower collision rate than the transdeltoid one and that a higher AI would identify shoulders in which transdeltoid nailing is unsafe.

## Materials and methods

All procedures were approved by the local ethics committee (EK-22-163-0922). As all datasets were fully anonymized, written informed consent was waived.

### CT data set

The institutional PACS was searched for shoulder CT examinations acquired between January 2020 and December 2024 for non-traumatic indications. Scans were obtained with patients supine, the forearm of the examined side resting across the abdomen. Clinical CT scans typically covered the shoulder without including the elbow; therefore, humeral torsion could not be quantified. Standard protocols used 120 kVp, collimation ≤ 1.25 mm and a bone or hybrid reconstruction kernel. After excluding prior surgery, deformity and radiographic osteoarthritis, 68 structurally normal shoulders from 57 adults were included.

### Three-dimensional model generation

DICOM image stacks were imported into Materialise Mimics (Materialise NV, Leuven, Belgium), whose combination of greyscale thresholding, region growing and manual editing permits selective extraction of cortical and cancellous structures that are required for accurate collision testing. The scapula and humerus were segmented independently, with primary emphasis on accurately reconstructing the cortical shell, and exported as surface meshes. Further processing was carried out in Materialise 3-matic (Materialise NV, Leuven, Belgium), which provides Boolean functions and a rigid-body collision detector for implant-bone interaction analysis without mesh simplification.

### Virtual nail design

A straight intramedullary nail was recreated in Autodesk Inventor (Autodesk Inc., San Rafael, CA, USA) from the original technical drawings of a widely used humeral nailing system. The smallest available diameter (8 mm) was selected to avoid conflating nail size with collision risk and to minimize the likelihood of supraspinatus footprint injury in clinical practice. The STL file was transferred to 3-matic for positioning.

### Definition of the optimal entry point and implant positioning

**Fig. 1:**
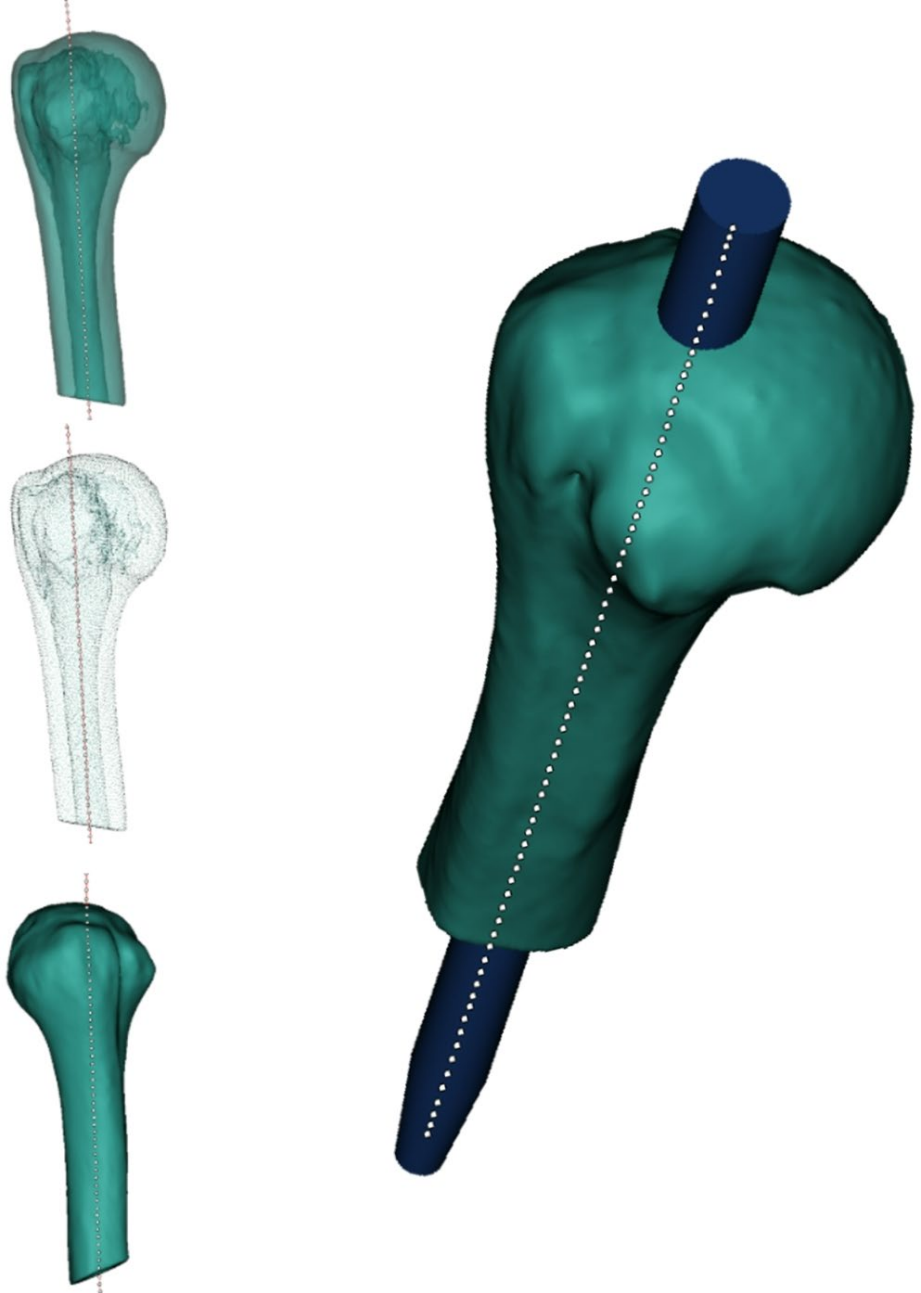
CT-based 3D modeling of the proximal humerus. The automated medullary centerline (dotted) marks the optimal nail entry point and implant insertion axis

The ideal entry site was defined as the point where a line collinear with the intramedullary canal intersects the articular surface of the humeral head while remaining at least 5 mm medial to the supraspinatus tendon insertion on the greater tuberosity. After marking this point, the nail model was aligned with the patient-specific medullary axis by best-fit registration and advanced until its proximal end coincided with the predefined entry site. The nail trajectory was defined as the extension of the diaphyseal centerline through the proximal humerus, corresponding to the intraoperatively targeted entry tract for straight antegrade nailing (Fig. 1).

### Simulation of shoulder motion and surgical approaches

The center of rotation was determined by fitting a sphere to the humeral head surface [20]. The humerus was rotated about its mediolateral axis, with the scapula kept static, to reproduce realistic arm positions. We modeled each approach with its clinically feasible intraoperative arm position: a strict transdeltoid in neutral (0°), where additional extension primarily rotates the head beneath the deltoid and requires non-physiologic traction, and an anterolateral portal with 30° extension, which reliably exposes the centrally located, tendon-sparing entry point anterior to the acromion in the beach-chair position.

**Fig. 2:**
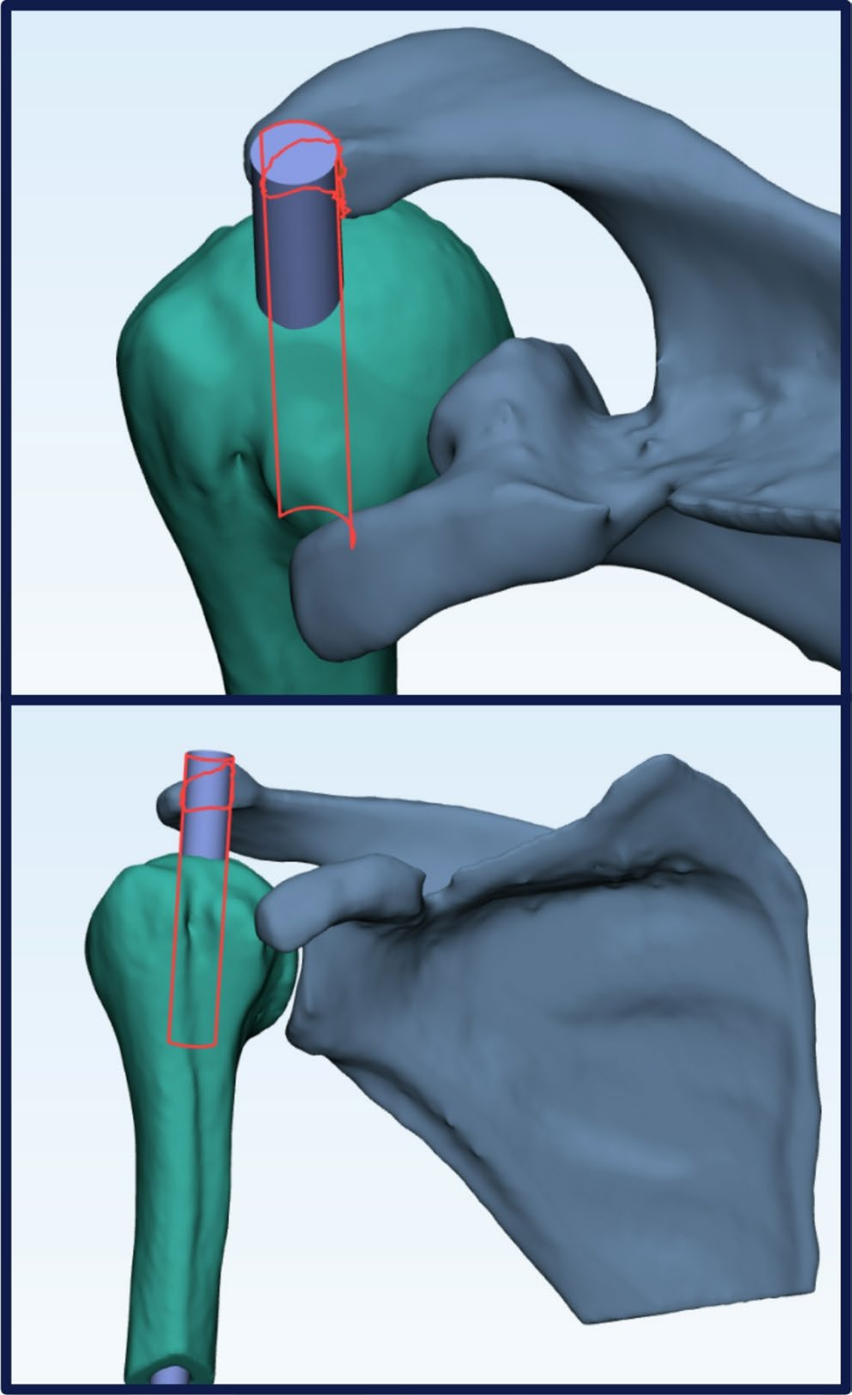
Automated collision‑detection output: the red overlay delineates the exact contact surface where the virtually inserted nail impinges on the acromion, allowing rapid, objective quantification of implant-bone interference

For each position, the collision algorithm recorded a binary outcome when any nail and acromion surface nodes intersected (Fig. 2).

### Acromion index measurement

The Acromion Index (AI) was calculated in an axial CT plane parallel to the glenoid face as the ratio of the glenoid-to-acromion distance (GA) to the glenoid-to-lateral-humerus distance (GH). An AI value closer to 1 indicates a more laterally extending acromion, while a value closer to 0 suggests a less prominent acromion. Although first described on standard radiographs, previous work has demonstrated equivalent reliability when the measurement is obtained from CT or MRI [21, 22].

### Observer workflow and reproducibility

Two investigators independently completed the entire workflow, from segmentation to collision scoring, while blinded to each other’s results. Agreement for the binary collision outcome was expressed with Cohen’s κ, and reproducibility of GA, GH and AI was assessed using two-way mixed intraclass correlation coefficients (ICC).

### Sample size and statistical analysis

A paired McNemar test was planned as the primary analysis. Power calculations indicated that at least 55 paired shoulders would be sufficient to detect a clinically relevant 20-percentage-point difference in collision rates between approaches (α = 0.05, power = 80%). The final sample of 68 shoulders exceeded this requirement. Binary logistic regression was used to examine whether the Acromion Index (AI) predicts collision during the transdeltoid approach, reporting odds ratios (OR) with 95% confidence intervals. Multivariable models adjusted for age (continuous, years), sex (binary), and side (left/right). To assess effect modification, interaction terms *AI × Age* and *AI × Sex* were tested; non-significant interactions were not retained in the final model. To contextualize generalizability, associations of AI with age were evaluated using Spearman’s rank correlation, and sex differences were tested using the Mann-Whitney U test. Exploratory comparisons of transdeltoid collision rates by sex and by side were performed using Fisher’s exact test; odds ratios with 95% confidence intervals were reported. Discrimination was quantified by receiver-operating-characteristic (ROC) analysis; the Youden index (J = TPR − FPR) defined the optimal AI threshold. For internal validation, a non-parametric bootstrap (B = 1000 resamples at the shoulder level) estimated the sampling distribution of AUC, Youden cut-off, sensitivity, specificity, PPV, and NPV; we report the bootstrap median and 95% percentile confidence intervals. Two-tailed p-values < 0.05 were considered significant.

This virtual workflow enabled a patient-specific, motion-aware comparison of the two most common surgical approaches for straight antegrade humeral nailing while controlling for implant diameter, entry point definition and observer variability.

## Results

The final cohort comprised 68 morphologically normal shoulders reconstructed from 57 anonymized CT examinations. Eleven patients had been scanned bilaterally, so shoulders outnumbered individuals. The mean age at imaging was 48.3 years with a broad spread across the adult lifespan (standard deviation 18.7, 95% confidence interval 44.2–52.4, absolute range 18.0–90.6 years). Only 15 of the 57 subjects were female, yielding a sex distribution of 73.7% male and 26.3% female. Side distribution was slightly left-weighted: 39 of 68 reconstructions (57.4%) represented left shoulders, 29 (42.6%) right shoulders.

Quantitative shoulder geometry is summarized in Table 1. Across all reconstructions the lateral reach of the acromion, expressed as the glenoid-acromion distance (GA), averaged 32.9 ± 4.1 mm, while the overall transverse breadth of the joint, measured as the glenoid-to-lateral-humerus distance (GH), averaged 49.4 ± 4.0 mm. Their ratio, the Acromion Index, centered on 0.668 with an inter-quartile range of 0.608 to 0.714, indicating that two-thirds of the shoulders placed the acromial edge roughly 67% of the way from the glenoid plane to the outer humeral cortex. None of the three metrics differed significantly between left and right shoulders (all *p* > 0.20), justifying pooled analysis. Measurement reliability was excellent: inter-observer intraclass correlation coefficients reached 0.91 for GA, 0.92 for GH and 0.90 for the derived AI, demonstrating that the landmark protocol can be repeated with minimal variance.

**Table 1:** Baseline morphometric measurements and inter-observer reliability (*n* = 68 shoulders) (SD = standard deviation; ICC = intraclass correlation coefficient; CI = confidence interval)

Metric	Mean ± SD	Median	Range (min - max)	Inter-observer ICC (95% CI)
Glenoid-Acromion distance (GA), mm	32.90 ± 4.11	33.10	22.4–45.2	0.91 (0.87–0.95)
Glenoid-Humerus distance (GH), mm	49.41 ± 4.04	49.50	41.9–57.9	0.92 (0.88–0.95)
Acromion Index (AI = GA/GH)	0.668 ± 0.082	0.676	0.50–0.93	0.90 (0.84–0.94)

Collision frequencies for each simulated access are detailed in Table 2. When the nail was introduced through the transdeltoid approach with the arm held in neutral, contact between the implant and the acromion occurred in 32 of 68 shoulders, a rate of 47.1%. Repeating the simulation through the anterolateral approach and adding 30° of glenohumeral extension lowered the collision count to two shoulders, corresponding to 2.9%. The paired McNemar analysis confirmed that this reduction was highly significant (χ² = 28.0, *p* < 0.001). The absolute risk difference of 44.1% translates into a number-needed-to-treat of 2.27.

**Table 2:** Implant-acromion collision by approach-specific standardized arm position (*p-value from paired McNemar test; CI = confidence interval)

Approach/Arm position	Collisions	Rate % (95% CI)	*p*-value vs. transdeltoid 0°*
Transdeltoid, 0° extension (reference)	32/68	47.1% (35.2–59.5)	-
Anterolateral, 30° extension	2/68	2.9% (0.7–11.3)	< 0.001

In exploratory subgroup analyses of the transdeltoid simulations, collision rates did not differ by sex (male 24/50 [48.0%] vs. female 8/18 [44.4%]; Fisher’s exact *p* = 1.00; OR = 1.15, 95% CI 0.39–3.41) or side (left 19/39 [48.7%] vs. right 13/29 [44.8%]; *p* = 0.81; OR = 1.17, 95% CI 0.45–3.07).

Logistic regression demonstrated that the Acromion Index was a powerful anatomical predictor of collision during the transdeltoid approach. Each increment of 0.10 in AI nearly tripled the odds of impingement (univariable OR = 3.17, 95% CI 1.52–6.59, *p* = 0.003), and the association persisted after adjustment for age, sex and laterality (adjusted OR = 2.81, *p* = 0.011). Receiver-operating-characteristic analysis produced an AUC of 0.72 (95% CI 0.60–0.83). The Youden criterion identified AI ≥ 0.69 as the best cut-off, yielding 63% sensitivity and 72% specificity, with a positive predictive value of 67% and a negative predictive value of 69%.

In additional analyses, the Acromion Index (AI) showed no significant correlation with age (Spearman ρ = 0.155; *p* = 0.207; *n* = 68) and no sex-specific differences (male median 0.676 vs. female 0.675; Mann-Whitney *p* = 0.939). A logistic model including AI, age, sex, and interaction terms (*AI × Age*, *AI × Sex*) revealed no significant interactions (both *p* > 0.70), indicating that the predictive effect of AI on transdeltoid collision is stable across age and sex. Bootstrap internal validation yielded an AUC of 0.726 (median; 95% CI 0.585–0.830) and a Youden-optimal AI threshold of 0.690 (median; 95% CI 0.597–0.732).

Methodological reliability was high throughout. Agreement between the two independent investigators reached a Cohen κ of 0.86 (95% CI 0.77–0.95) for the binary collision outcome, while mean absolute differences for GA and GH measurements were 0.7 mm and 0.9 mm, respectively, amounts that represent less than 2% of their means.

The study was also adequately powered: the final sample of 68 paired shoulders exceeded the 55 pairs calculated a priori to detect a 20-percentage-point difference between approaches with 80% power at a two-sided α of 0.05, ensuring that the observed effects are unlikely to be due to chance fluctuation.

## Discussion

This patient-specific CT simulation shows that acromial interference during straight antegrade humeral nailing depends mainly on the chosen surgical approach and the respective arm position. In neutral alignment the transdeltoid approach produced a collision in 47.1% of simulations, whereas the anterolateral approach with 30° extension lowered the rate to 2.9%. An absolute risk reduction of 44.1% translates into a number-needed-to-treat of 2.27; thus, treating just over two patients with the anterolateral rather than the transdeltoid approach prevents one implant-acromion collision.

Our findings should be interpreted as evaluating each surgical approach together with its clinically permitted positioning, rather than the approach in isolation. The lower collision rate observed with the anterolateral portal reflects the combined effect of its anterior working corridor and the physiologically obtainable 30° of extension, which together enlarge the safe window to the centrally located, tendon-sparing entry point without forceful retraction. Because the feasible degree of extension differs by approach, the extension angle is approach-inherent in our model and mirrors the intraoperative decision surgeons actually face. Consequently, our results speak to approach-with-positioning, not to an artificial, position-matched comparison that would require non-physiologic traction for a strict transdeltoid approach.

A correctly centered entry point is critical for fracture fixation stability [23, 24]. If it is placed too far lateral in order to bypass the acromion, the proximal nail ends up within the greater tuberosity where cancellous density is markedly lower than in the subchondral apex [14]. Euler et al. termed the optimal, centrally positioned entry point as the “fifth anchoring point”, highlighting its role in resisting varus collapse. The name refers to the fact that a straight antegrade nail already secures the fracture with four conventional points of purchase – two distal locking screws, one calcar screw and the lateral locking set screw in the nail head. Seating the nail crown in dense subchondral bone at the humeral-head apex adds a fifth point of fixation, creating a compressive buttress against varus bending and markedly increasing resistance to collapse [16, 17]. Our results underscore that this apical anchorage should be preserved; surgeons should therefore choose an approach that exposes the true head apex instead of diverting the nail through low-density tuberosity bone.

Earlier clinical investigations acknowledged that a prominent acromion may hinder coaxial nail insertion by physically obstructing the entry point [13]. However, no prior study has systematically quantified the biomechanical benefit of controlled humeral extension or assessed acromial clearance across multiple surgical approaches in a paired, motion-aware setting. Many authors emphasized the importance of a collinear entry point medial to the supraspinatus footprint but did not evaluate the role of acromial overhang in obstructing nail insertion [16, 25, 26].

A safe nail trajectory must pass medial to the supraspinatus footprint while remaining coaxial with the humeral canal. With a strictly lateral, transdeltoid approach these two demands clash more often as the acromion projects farther laterally, because the entry point cannot be advanced anteriorly. Bringing the arm into adduction might theoretically open the view, but the torso blocks the movement, and the entry zone remains obscured. By contrast, the anterolateral approach lies in front of the acromion. Extending the shoulder by approximately 30° moves the humeral head anteriorly and brings the central, tendon-sparing entry point into the field without forceful deltoid retraction [11, 12, 27].

In our model, the nail trajectory is anchored to the diaphyseal centerline; therefore, interindividual differences in humeral torsion would rotate the tract about its axis rather than shift its surface projection relative to the acromion, making any influence on implant-acromion collision minor compared with the approach corridor and achievable shoulder extension. Although torsion could not be quantified due to the CT field-of-view (elbow not included), all scans were obtained in a standardized arm position, and intraoperative internal/external rotation can be adjusted if required.

Clinically the results support a patient-specific algorithm. Shoulders with an Acromion Index of 0.69 or higher are better treated through the anterolateral approach with moderate extension. Although shoulders below that value often tolerate a transdeltoid split, many surgeons may still prefer the anterolateral approach because it consistently enlarges the working space at minimal cost. The proposed threshold should be viewed as a first guideline that requires confirmation in broader, especially osteoporotic, cohorts.

Strengths of the study include a paired design that removes interindividual variability, high resolution segmentation, excellent inter-observer reliability, and the first link between a simple planar index and a fully three-dimensional, motion-aware outcome. Limitations are the younger, male-predominant cohort, lack of humeral torsion correction and bone quality modelling, and the virtual nature of the analysis. Because feasible arm extension differs by approach, the extension angle is inherent to the approach in our model; while this reflects real-world practice, it precludes isolating the effect of ‘approach’ under strictly identical positioning.

Subgroup analyses were underpowered for the anterolateral approach due to only two observed collisions; moreover, available evidence suggests bilateral symmetry of relevant shoulder anatomy and side-invariant acromial metrics, which is consistent with our null finding for side [28, 29]. Humeral torsion was not measured due to the limited CT field-of-view; while extreme torsional variants could theoretically influence tract orientation, we expect only a minor impact on acromial clearance given the centerline-based trajectory and the ability to adjust rotation intraoperatively.

Our cohort overrepresents younger male individuals due to exclusion criteria (prior surgery, deformity, radiographic osteoarthritis) aimed at isolating anatomical effects. Nevertheless, within-cohort analyses and external evidence suggest that the Acromion Index (AI) is not materially influenced by age or sex. Specifically, we observed no association between AI and age and no sex-related differences; prior studies likewise report no sex or age differences in AI and no age-related acromial changes in large imaging and cadaveric series [29–31]. Moreover, while osteoporosis widens the proximal humeral canal with age, it does not affect the canal centerline and therefore is unlikely to change the nail entry trajectory [32]. Accordingly, the AI-based guidance and the observed reduction of acromion collision with the anterolateral approach are likely generalizable to older/osteoporotic patients; still, we acknowledge that prospective validation in an elderly osteoporotic cohort is warranted, and we have tempered our conclusions accordingly.

Future work should validate the simulation prospectively, automate segmentation with deep learning, and examine whether raw data from the uninjured shoulder can be mirrored for truly personalized plans. Adding bone density maps and deformable cuff models would extend predictions from geometric feasibility to fixation strength and rotator cuff safety, closing the gap between virtual rehearsal and surgical reality.

## Conclusion

Our patient-specific simulation shows that straight antegrade nailing performed through an anterolateral approach in 30° extension almost abolishes implant-acromion collision, cutting the rate from 47% to 3%. Given this 15-fold safety margin, routine use of the transdeltoid split is no longer justified; the anterolateral approach should become the default. An Acromion Index ≥ 0.69 remains a practical flag for cases in which a lateral entry is particularly unsafe, yet our data suggest choosing the anterior access even below that threshold. The semi-automated simulation framework itself offers a versatile tool for future patient-specific evaluation of other surgical techniques and implants.

## Data Availability

Anonymized datasets generated during the study can be obtained from the corresponding author on reasonable request.
